# Relationship between Physical Activity and Plasma Fibrinogen Concentrations in Adults without Chronic Diseases

**DOI:** 10.1371/journal.pone.0087954

**Published:** 2014-02-03

**Authors:** Manuel A. Gomez-Marcos, José I. Recio-Rodríguez, Maria C. Patino-Alonso, Vicente Martinez-Vizcaino, Carme Martin-Borras, Aventina de-la-Cal-dela-Fuente, Ines Sauras-Llera, Alvaro Sanchez-Perez, Cristina Agudo-Conde, Luis García-Ortiz, on behalf of the EVIDENT Study Investigators

**Affiliations:** 1 Primary Care Research Unit, The Alamedilla Health Center, Castilla and León Health Service–SACYL, IBSAL, and Department of Medicine, University of Salamanca, Salamanca, Spain; 2 Primary Care Research Unit, The Alamedilla Health Center, Castilla and León Health Service–SACYL, Salamanca, Spain; 3 Stadistics Department, University of Salamanca, and Primary Care Research Unit, The Alamedilla Health Center, Salamanca, Spain; 4 Social and Health Care Research Center, University of Castilla-La Mancha, Cuenca, Spain; 5 DEA, Ciències de l'Educació i l'Esport (FPCEE) Universitat Ramon Llull, Barcelona, Spain; 6 Casa of Barco Health Center, Castilla and León Health Service–SACYL,Valladolid, Spain; 7 Torre Ramona Health Center, Aragón Health Service – Salud, Zaragoza, Spain; 8 Primary Care Research Unit of Bizkaia, Basque Health Service-Osakidetza, Bilbao, Spain; Universidad Pablo de Olavide, Centro Andaluz de Biología del Desarrollo-CSIC, Spain

## Abstract

**Objective:**

To analyze the relationship between regular physical activity, as assessed by accelerometer and 7-day physical activity recall (PAR), and plasma fibrinogen concentrations.

**Methods:**

A cross-sectional study in a previously established cohort of healthy subjects was performed. This study analyzed 1284 subjects who were included in the EVIDENT study (mean age 55.0±13.6 years; 60.90% women). Fibrinogen concentrations were measured in blood plasma. Physical activity was assessed with a 7-day PAR (metabolic equivalents (METs)/hour/week) and GT3X ActiGraph accelerometer (counts/minute) for 7 days.

**Results:**

Physical exercise, which was evaluated with both an accelerometer (Median: 237.28 counts/minute) and 7-day PAR (Median: 8 METs/hour/week). Physical activity was negatively correlated with plasma fibrinogen concentrations, which was evaluated by counts/min (r = −0.100; p<0.001) and METs/hour/week (r = −0.162; p<0.001). In a multiple linear regression analysis, fibrinogen concentrations of the subjects who performed more physical activity (third tertile of count/minute and METs/hour/week) respect to subjects who performed less (first tertile), maintained statistical significance after adjustments for age and others confounders (β = −0.03; p = 0.046 and β = −0.06; p<0.001, respectively).

**Conclusions:**

Physical activity, as assessed by accelerometer and 7-day PAR, was negatively associated with plasma fibrinogen concentrations. This relation is maintained in subjects who performed more exercise even after adjusting for age and other confounders.

## Introduction

Participation in regular physical activity and/or aerobic exercise training is associated with a decrease in all-cause, cardiovascular and cancer mortality [1]–[3] and a reduced risk of fatal and non-fatal coronary events in healthy individuals over a wide age range [4]. A sedentary lifestyle is one of the major risk factors for cardiovascular disease [5].

The anti-inflammatory effects of exercise are thought to be mechanisms that explain the well-documented cardioprotective effects of physical activity [6]–[8]. Regular physical activity is associated with lower levels of inflammation markers over 10 years of follow-up [9].

The meta-analysis by Kaptoge et al. [10], which included 33 studies with 883372 participants, concluded that plasma fibrinogen concentrations were associated with cardiovascular and non-cardiovascular mortality, although its effects faded when adjusted for other cardiovascular risk factors. They also found that long-term increases in plasma fibrinogen concentrations of 1 g/litre were associated with an approximate doubling of the risk for major cardiovascular disease outcomes. Similarly, the addition of fibrinogen concentrations to classic risk factors used in assessment scales for cardiovascular risk in people with an intermediate risk can help predict additional events over a 10-year time period [11].

However, studies that have examined the relationship of physical activity with fibrinogen have generally been conducted by subjectively measuring physical activity through questionnaires completed by the subjects. Objective assessments by an accelerometer and the relationship of this measure with fibrinogen in the general population have not yet been studied.

We hypothesized that physical activity can modify plasma fibrinogen concentrations. Therefore, we aimed to analyze the relationship between physical activity assessed objectively by an accelerometer and self-reported physical activity through 7-day physical activity recall (PARs) with plasma fibrinogen.

## Methods

The 1284 subjects analyzed in this work derive from the EVIDENT study (NCT01083082) [12].

### Study population

Patients ranging 20–80 years of age were selected through random sampling from general practitioner offices in 6 health centers. The exclusion criteria were the following: known coronary or cerebrovascular atherosclerotic disease, heart failure, moderate or severe chronic obstructive pulmonary disease, walk-limiting musculoskeletal disease, advanced respiratory, advanced respiratory, renal, or hepatic disease; severe mental disease; treated oncological disease diagnosed in the past 5 years; terminally ill patients; and pregnant women.

Of the 1553 subjects included in the EVIDENT study, 169 were excluded because they did not have plasma fibrinogen concentrations (115) or accelerometer (154) measurements. Therefore, this study analyzed 1284 subjects in total. The sample size calculation indicated that the number of patients included in the study (1284) was sufficient to detect a difference of 20 mg/dl of fibrinogen among tertiles of Counts/minute in a two-sided test, assuming a common standard deviation (SD) of 89 mg/dl in fibrinogen with a significance level of 5% and a power of 80%. The study was approved by an independent ethics committee of Salamanca University Hospital (Spain), and all participants gave written informed consent according to the general recommendations of the Declaration of Helsinki [13].

### Anthropometric measurements

Body weight was determined on two occasions using a homologated electronic scale (Seca770; Medical scale and measurement systems, Birmingham, United Kingdom) following due calibration (precision ±0.1 kg), with the patient wearing light clothing and shoeless. These readings were rounded to 100 g. Height in turn was measured with a portable system (Seca 222; Medical scale and measurement systems, Birmingham, United Kingdom), recording the average of two readings, and with the patient shoeless in the standing position. The values were rounded to the closest centimeter. Body mass index (BMI) was calculated as weight (kg) divided by height squared (m^2^). Waist circumference was measured using a flexible graduated measuring tape with the patient in the standing position without clothing. The upper border of the iliac crests was located, and the tape was wrapped around above this point, parallel to the floor, ensuring that it was adjusted without compressing the skin. Body fat percentage was measured using a body fat monitor previously validated (OMRON, model BF306; Omron Health Care, Kyoto, Japan) [14].

### Laboratory determinations

Venous blood sampling was performed between 08:00 and 09:00 hours after the individuals fasted and abstained from smoking and the consumption of alcohol and caffeinated beverages for the previous 12 hours. Blood samples were collected in the respective health centers, and were analyzed at the hospital of the city participating in external quality assurance programs of the Spanish Society of Clinical Chemistry and Molecular Pathology. Fasting plasma glucose, creatinine, uric acid, serum total cholesterol, HDL-cholesterol and triglyceride concentrations were measured using standard enzymatic automated methods. LDL cholesterol was estimated by the Friedewald equation when the direct parameter was not available. Glycated haemoglobin was measured with an immune-turbidimetric assay. High sensitive C-reactive protein levels and fibrinogen concentrations were determined by immunoturbidimetric assay. The blood concentration of insulin was determined by chemiluminescent microparticle immunoassay. The insulin sensitivity was determined with the Homeostasis Model Assessment Insulin Resistance (HOMA-IR) index [15] through the following formula: fasting glucose (mmol/l) × fasting insulin (mU/ml)/22.5.

### Office or clinical blood pressure

Office blood pressure measurement involves three measurements of systolic blood pressure (SBP) and diastolic blood pressure (DBP), using the average of the last two, with a validated OMRON model M7 sphygmomanometer (Omron Health Care, Kyoto, Japan), and following the recommendations of the European Society of Hypertension [16]. The mean blood pressure (MBP) was calculated by the following equation: MBP  =  [(2 * DBP) + SBP]/3.

### Physical activity

Physical activity was estimated by the 7-day physical activity recall (PAR) and an accelerometer. The 7-day PAR is a general measure of physical activity, which has been recognised as a valid and reliable tool in recent years and is widely used in epidemiological, clinical and behavioural change studies [17]. It consists of a semi-structured interview (10–15 minutes) in which participants provide an estimate of the number of hours dedicated to physical or occupational activities that required at least a moderate effort over the previous 7 days. The categories collected are the following: moderate, hard and very hard physical activity. The amount of time dedicated to each activity is multiplied by the mean metabolic equivalents (METs) of each category, as follows: light activity  = 1.5; moderate  = 4; hard  = 6; and very hard  = 10. The sum of the products of the hours dedicated to each activity and its estimated mean energy expenditure provides an estimation of the kilocalories per kilogram used per day (kcal/kg/d). The dose of physical activity was estimated in METs/hour/week, and active persons were considered as those doing at least 30 minutes of moderate activity for five days a week or at least 20 minutes of hard activity for 3 days a week. Persons not reaching this level of physical activity were considered sedentary [18].

ActiGraph GT3X accelerometers (ActiGraph, Shalimar, FL, USA) were used, which have been previously validated [19]. Subjects wore the accelerometer fastened with an elastic strap to the right side of the waist for 7 consecutive days except for during bathing and performing activities in water. The data were recorded at 1-minute intervals. The total physical activity was expressed in counts per minute. The intensity of the physical activity (low, moderate or high) was determined according to the cut-off points proposed by Freedson [20]. When evaluated the accelerometer data, three segments of the day were defined: morning (8 am to 3 pm), afternoon (3 pm to 10 pm) and evening (10 pm to 8 am).

Reliability between 7-day PAR and accelerometer data was evaluated by correlation coefficient (CC) between METs/hour/week and counts/minute (r = 0.397; p<0.001) and total energy expenditure measured by both methods (r = 0.471 p<0.001) and intraclass CC of total energy expenditure (r = 0.584 (95% CI: 0.493 to 0.660); p<0.001). Convergent validity was evaluated through the correlation between both tools used to measure physical activity with the body fat percentage, counts/min (r = −0.230; p<0.001) and METs/hour/week (r = −0.286; p<0.001).

### Statistical analysis

The continuous variables were expressed as the mean ± standard deviation for normally distributed continuous data, medians (interquartile range) for asymmetrically distributed continuous data and frequency distribution for categorical data. Statistical normality was checked using the Kolmogorov–Smirnov test.

A Spearman's correlation and intra-class correlation coefficients were used to analyze the relationship between asymmetrically distributed continuous data.

For the comparison in fibrinogen values between different groups (tertiles) according to its physical activity levels (counts/min and METS/hour/week), the non parametric test (Kruskal Wallis) and post hoc comparisons were used. We performed a multiple linear regression analysis, considering the plasma fibrinogen concentrations as dependent variable and physical activity as independent variable. The plasma fibrinogen concentrations do not follow a normal distribution and it has been subjected to log transformation in order to estimate the regression model. Physical activity has been analyzed by considering their distribution in tertiles of counts/minute and METs/hours/week (1st tertile less exercise and 3rd tertile the most exercise). We adjusted the models in a second step for age and gender (male  = 1/female  = 0) and in a third step for ln waist circumference, alcohol, total cholesterol, lipid lowering drugs (yes  = 1; no  = 0) and mean blood pressure. The data were analyzed using IBM® SPSS® v.20 software. A value of p<0.05 was considered statistically significant.

## Results

We studied 1284 subjects with a mean age of 55.0±13.6 years, of which 60.90% were female. Table 1 shows the demographic and clinical characteristics of the study patients.

**Table 1:** Baseline characteristics of participants in EVIDENT study.

	Mean ± SD/Median (IQR)/n (%)
Age (years)	55.0±13.6
Gender n (%)	
Male	502 (39.10)
Female	782 (60.90)
Smoking status n (%)	
Never	613 (47.70)
Current	276 (21.50)
Past	395 (30.80)
Alcohol. (g/week)	10 (70-0)
Body mass index (kg/m^2^)	26.7 (24.0–29.5)
Waist circumference cm	93 (85.00–100.75)
Obesity n (%)	278 (21.70)
Abdominal Obesity n (%)	560 (43.80)
Body fat percentage (%)	34.80±7.50
Waist/Height cm/cm	0.56 (0.52–0.61)
Body adiposity index	26.0 (22.2–30.2)
Office systolic blood pressure (mmHg)	124.7±17.0
Office diastolic blood pressure (mmHg)	77.3±10.6
Mean blood pressure (mmHg)	92.9±11.5
Hypertension n (%)	357 (28.00)
Fasting glucose (mg/dL)	89 (82.50–98.00)
Glycated hemoglobin (%)	5.50 (5.30–5.80)
HOMA Index	1.52 (0.86–2.43)
Diabetes n (%)	96 (7.50)
Total cholesterol (mg/dL)	214.29±38.44
Triglycerides (mg/dL)	96 (71.00–135.00)
HDL-cholesterol (mg/dL)	58.00 (48.75–69.00)
LDL-cholesterol (mg/dL)	133.15±34.65
Dyslipidemia n (%)	384 (30.10)
Lipid lowering drugs n (%)	238 (18.50)
Creatinine. (mg/dL)	0.8 (0.70–0.90)
Haemoglobin (g/dL)	14.30 (13.50–15.30)
Fibrinogen (mg/dL)	366 (319.00–415.00)
Ln Fibrinogen (mg/dL)	5.90±0.22
hs-CRP (mg/dL)	0.17 (0.09–0.34)
Uric Acid (mg/dL)	4.80 (3.90–5.90)

Values are means (standard deviations) for continuous data. Median (interquartile range) for asymmetrically distributed continuous data and number and proportion for categorical data.

Body adiposity index  =  (hip circumference in centimeters)/(height in meters)^1.5^ − 18); HDL: High Density Lipoprotein; LDL: low-density lipoprotein; hs-CRP: high-sensitivity C-Reactive Protein.

Table 2 shows the physical activity as measured by both an accelerometer (counts/minute) and a 7-day PAR (METs/hour/week).

**Table 2:** Physical exercise assessment by accelerometer and 7-day PAR.

Accelerometer	Median/n	IQR/(%)
Counts/minute	237.28	177.93–306.68
Sedentary (minutes/day)	1057.12	1012.30–1119.79
Light (minutes/day)	327.79	275. 97–363.47
Moderate (minutes/day)	45.14	27.26–65.62
Hard and very hard (minutes/day)	0.00	0.00–0.45
Kilocalories/day (KCALS)	241.53	130.07–402.67
**7- day PAR**		
METs/hour/week	8	0.00–25.41
**Active/sedentary according 7-par day**		
Sedentary n (%)	879	68.20
Active n (%)	405	31.80

Values are medians and interquartile range(IQR) for asymmetrically distributed continuous data and number and proportions for categorical data.

METs: metabolic equivalent. Active were considered as those doing at least 30 minutes of moderate activity, five days a week, or at least 20 minutes of hard activity, 3 days a week.

Subjects performed more physical activity in the morning (120.30 counts/min) than in other segments of the day (afternoon: 100.13 counts/min, evening: 16.85 counts/min).

Physical activity, as assessed by counts/minute and METs/hour/week showed an inverse correlation with the plasma fibrinogen concentrations (r = −0.100; p<0.001 r = −0.162; p<0.001 respectively) and with all anthropometric parameters. The correlation of physical activity with other biological parameters is shown in Table 3. Time expended in heavy/very heavy physical activity was inversely associated with the plasma fibrinogen concentrations (r = −0.116; p<0.01).

**Table 3:** Bivariate correlations of physical exercise (Counts/minute and METS/hour/week) with biological and anthropometric parameters.

	Count/minute	METS/Hour/week
Fibrinogen (mg/dL)	−0.100**	−0.162**
hs-CRP (mg/dL)	−0.181*	−0.179*
HOMA Index	−0.137**	0.055
Creatinine (mg/dL)	0.007	0.162**
Albumin/creatinine index (mg/g)	−0.045	−0.049
Uric Acid (mg/dL)	−0.026	0.106**
Glycated hemoglobin (%)	−0.085**	0.023
Haemoglobin (mg/dL)	0.075**	0.084**
Total Cholesterol (mg/dL)	0.038	−0.009
HDL-Cholesterol (mg/dL)	0.133**	0.019
LDL-Cholesterol (mg/dL)	0.044	−0.015
Triglycerides (mg/dL)	−0.173**	−0.063*
Body mass index (kg/m^2^)	−0.126**	−0.081**
Body fat percentage (%)	−0.230**	−0.236**
Waist/Height cm/cm	−0.161**	−0.111**
Body adiposity index	−0.173**	−0.155**

Counts/minute by accelerometer; METS/hour/week by 7-Par/day; hs-CRP: high-sensitivity C-reactive protein; HDL: high-density lipoprotein; LDL: low-density lipoprotein; Body adiposity index =  ((hip circumference)/((height)^1.5^) -18)).

P-values by Spearman correlation. * p<0.05 ** p<0.01.

In a multiple linear regression analysis, which considered fibrinogen as dependent variable (Table 4), and the counts/minute tertiles as independent variable. Fibrinogen concentrations of the subjects who performed more physical activity (third tertile) respect to subjects who performed less (first tertile), maintained the statistical significance in all three models, with the first model unadjusted (β = −0.06; p<0.001), the second model adjusted for age and sex (β = −0.04; p<0.001) and the third model adjusted for age, sex, waist circumference, alcohol consumption, total cholesterol, hypolipidaemic drugs and mean arterial pressure (β = −0.03; p = 0.046). Similarly with the METs/hour/week tertiles, fibrinogen concentrations of the subjects who performed more physical activity (third tertile) respect to subjects who performed less (first tertile) maintained statistical significance in all three models. In the first model unadjusted (β = −0.08; p<0.001), the second model adjusted for age and sex (β = −0.06; p<0.001) and the third model adjusted for age, sex, waist circumference, alcohol consumption, total cholesterol, hypolipidaemic drugs and mean arterial pressure (β = −0.06; p<0.001).

**Table 4:** Multiple regression with plasma fibrinogen concentrations as dependent variable and physical activity as independent variable.

Dependent Variable: Ln_Fibrinogen	β	95% CI		p	Dependent Variable: Ln_Fibrinogen	β	95% CI		p
**Model 1**									
Counts/minute (T1)					METS/hours/week (T1)				
Counts/minute (T2)	−0.03	−0.06	0.01	0.061	METS/hours/week (T2)	−0.04	−0.07	−0.01	0.010
Counts/minute (T3)	−0.06	−0.08	−0.02	<0.001	METS/hours/week (T3)	−0.08	−0.11	−0.05	<0.001
**Model 2**									
Counts/minute (T1)					METS/hours/week (T1)				
Counts/minute (T2)	−0.02	−0.05	0.02	0.189	METS/hours/week (T2)	−0.03	−0.07	−0.01	0.028
Counts/minute (T3)	−0.04	−0.07	−0.02	0.008	METS/hours/week (T3)	−0.06	−0.10	−0.04	<0.001
Sex	−0.07	−0.09	−0.05	<0.001	Sex	−0.06	−0.09	−0.04	<0.001
Age	0.10	0.06	0.15	<0.001	Age	0.11	0.07	0.15	<0.001
**Model 3**									
Counts/minute (T1)					METS/hours/week (T1)				
Counts/minute (T2)	−0.02	−0.05	0.01	0.268	METS/hours/week (T2)	−0.03	−0.07	−0.01	0.035
Counts/minute (T3)	−0.03	−0.06	−0.01	0.046	METS/hours/week (T3)	−0.06	−0.09	−0.03	<0.001
Sex	−0.08	−0.11	−0.06	<0.001	Sex	−0.08	−0.11	−0.05	<0.001
Age	0.07	0.02	0.11	0.007	Age	0.07	0.02	0.12	0.004
Waist circumference. (cm)	0.20	0.10	0.31	0.001	Waist circumference	0.20	0.10	0.31	<0.001
Alcohol (g/week)	0.00	0.00	0.00	0.115	Alcohol (g/week)	0.00	0.00	0.00	0.127
Total cholesterol. (mg/dL)	0.00	0.00	0.00	0.592	Total cholesterol. (mg/dL)	0.00	0.00	0.00	0.711
Lipid lowering drugs	0.02	−0.02	0.05	0.333	Lipid lowering drugs	0.01	−0.02	0.05	0.388
Mean blood pressure (mmHg)	0.01	0.00	0.01	0.209	Mean blood pressure (mmHg)	0.01	0.00	0.01	0.258

Dependent Variable: Ln_Fibrinogen

Independent Variables: Tertiles of Counts/minute. Tertiles of METS/hours/week.

Adjusted variables: Sex (1: male; 0: female); lnAge; Waist circumference. (cm); Alcohol (g/week); Total cholesterol. (mg/dL); Lipid lowering drugs (1:yes; 0:no); Mean blood pressure (mmHg)

**Figure 1:**
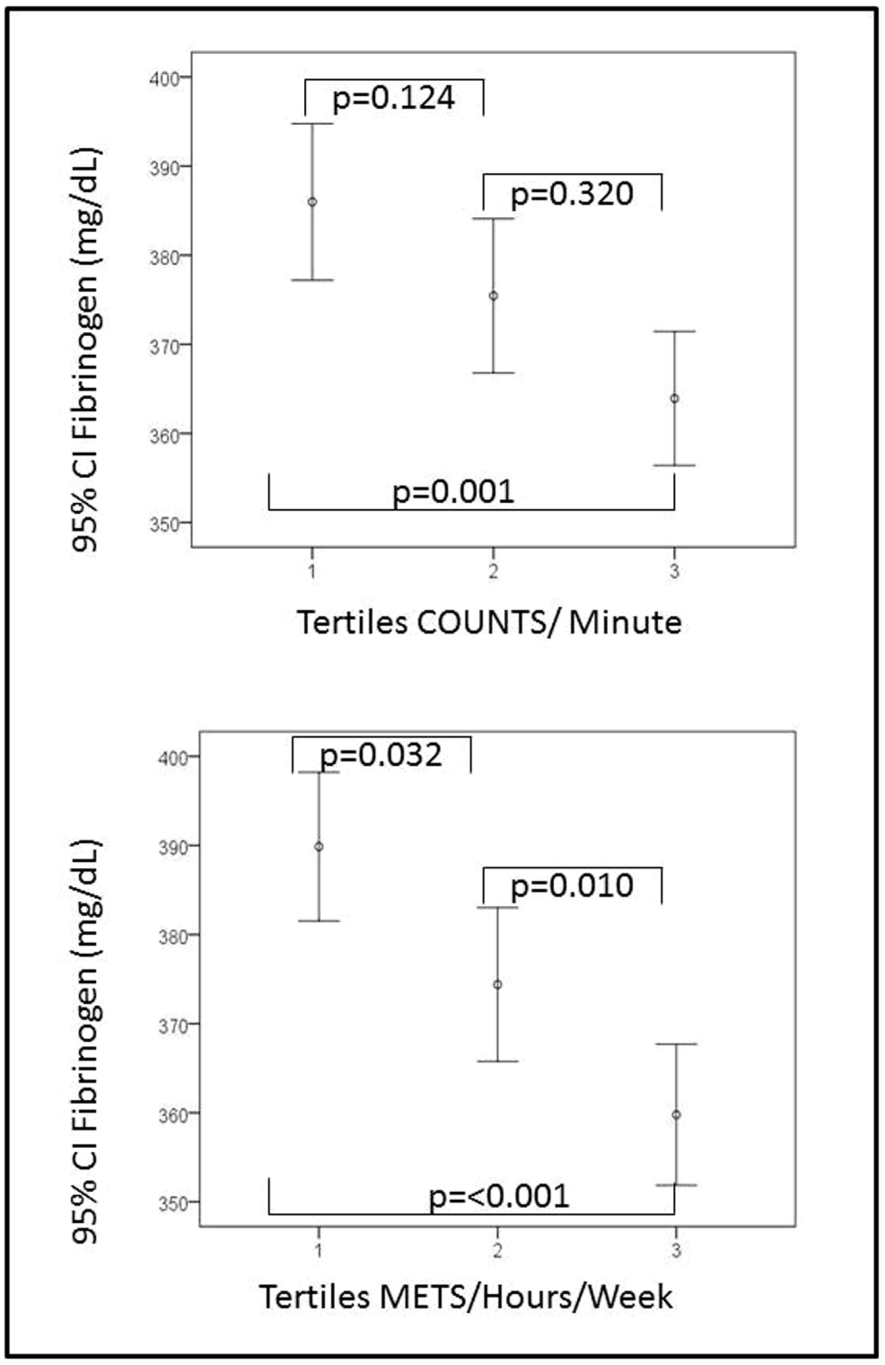
Plasma Fibrinogen concentrations according to the counts/minute and METs/hour/week tertiles. Count/minute tertiles (T1<195; T2 195 to 278; T3>278). METS/hours/week tertiles (T1<1; T2 1 to 17.5; T3>17.5). P-values by Kruskal-Wallis test: a) counts/min p<0.001; b) METS/hour/week p<0.05.

Figure 1 shows the mean values of plasma fibrinogen concentrations according to the counts/minute tertiles with significant differences between the first and the third tertile (p<0.001). The mean values of plasma fibrinogen concentrations according to the METs/hour/week tertiles showed statistical significance between the three tertiles (p<0.05, all).

## Discussion

According to the results of this study, physical activity was inversely associated with plasma fibrinogen concentrations, as assessed by both an accelerometer and a 7-day PAR. These associations were maintained in a multiple regression analysis after adjustments for other confounding factors in subjects in the third tertile of physical activity. This reinforces the importance of the quantity and intensity of physical activity in reducing the inflammatory cascade and of assessment factors such as fibrinogen that are used in the clinic.

The fact that individuals performed more physical activity in the morning than in other segments of the day can be useful in future intervention designs to promote physical activity.

The assessment of physical activity by questionnaires has often been questioned due to the influence of the individual's subjective perception. In this study, physical activity was assessed with two tools for one week, a subjective one (the 7-day PAR), in which the subject self-reported activity, and an objective one, which was measured with an accelerometer. The inverse relationship between physical activity and plasma fibrinogen concentrations that was obtained in this study was similar using both tools. This supports the validity of the physical activity data reported by the subject in the 7-day PAR and is consistent with the data published in other studies [6], [21]–[23].

Observational studies show that the anti-inflammatory effects of exercise are stable and that physical activity decreases fibrinogen values [6], [8], [21], which coincides with the results of this work. Similarly, the reduction of inflammatory markers by performing physical exercise is as effective as that achieved with drug therapy, suggesting that exercise may have broad anti-inflammatory effects [1].

However, the study results published in the National Health and Nutrition Examination Survey (NHANES) III study [21] suggest that inflammatory markers are lower only in subjects who perform vigorous activity (running and aerobic exercise), compared with those who perform a less intense exercise, which supports the potential importance of the exercise intensity in decreasing inflammation. Studies evaluating the association of physical activity and mortality found that after adjustments for confounders, this association was only maintained in the groups that performed a moderate intensity exercise HR  = 0.54, (95% CI  = 0.41–0.72) [2]. In the Baltimore study, which was conducted in a generally healthy population, the changes in total and high intensity physical activity were independent predictors of all-cause mortality only in men [24].

To summarise, these results indicate the importance of considering both the intensity and the duration of physical activity when examining the relationship between exercise and other inflammatory factors and mortality. Similarly, Kirk A. et al. [25] concluded according to the results of one intervention to increase the physical activity in type 2 diabetics, that the physical activity level was inversely associated with the plasma fibrinogen concentrations (p = 0.03).

### Limitations

The main limitation was the source of the data for the cross-sectional study, which prevented us from establishing a temporal relationship between the activity assessed with the accelerometer or 7-day PAR and the plasma fibrinogen concentrations for one week. In addition, we do not have physical activity monitoring data at an individual level; therefore, we must assume that the physical activity patterns remain fairly stable over time. However, this is the first study that examined the relationship between physical activity measured with two tools and plasma fibrinogen concentrations in a sample of patients from a multicentre study who were randomly selected so that they represented the patients visiting the primary care clinics of different territories of the Spanish state.

## Conclusions

Physical activity, as assessed by accelerometer and 7-day PAR, was inversely associated with plasma fibrinogen concentrations. This relation was maintained in subjects who performed more exercise even after adjusting for age and other confounders. Further research prospective/intervention studies are required in order to confirm the inverse association found between the physical activity and plasma fibrinogen concentrations.
